# Targeting autocrine HB-EGF signaling with specific ADAM12 inhibition using recombinant ADAM12 prodomain

**DOI:** 10.1038/srep15150

**Published:** 2015-10-19

**Authors:** Miles A. Miller, Marcia L. Moss, Gary Powell, Robert Petrovich, Lori Edwards, Aaron S. Meyer, Linda G. Griffith, Douglas A. Lauffenburger

**Affiliations:** 1 Massachusetts Institute of Technology, Department of Biological Engineering, 77 Massachusetts Ave., Cambridge, MA 02139; 2 BioZyme, Inc., 1513 Old White Oak Church Road, Apex, NC 27523; 3 National Institutes of Environmental Health Services (NIEHS), 111 TW Alexander Dr. RTP, NC 27709.

## Abstract

Dysregulation of ErbB-family signaling underlies numerous pathologies and has been therapeutically targeted through inhibiting ErbB-receptors themselves or their cognate ligands. For the latter, “decoy” antibodies have been developed to sequester ligands including heparin-binding epidermal growth factor (HB-EGF); however, demonstrating sufficient efficacy has been difficult. Here, we hypothesized that this strategy depends on properties such as ligand-receptor binding affinity, which varies widely across the known ErbB-family ligands. Guided by computational modeling, we found that high-affinity ligands such as HB-EGF are more difficult to target with decoy antibodies compared to low-affinity ligands such as amphiregulin (AREG). To address this issue, we developed an alternative method for inhibiting HB-EGF activity by targeting its cleavage from the cell surface. In a model of the invasive disease endometriosis, we identified A Disintegrin and Metalloproteinase 12 (ADAM12) as a protease implicated in HB-EGF shedding. We designed a specific inhibitor of ADAM12 based on its recombinant prodomain (PA12), which selectively inhibits ADAM12 but not ADAM10 or ADAM17. In endometriotic cells, PA12 significantly reduced HB-EGF shedding and resultant cellular migration. Overall, specific inhibition of ligand shedding represents a possible alternative to decoy antibodies, especially for ligands such as HB-EGF that exhibit high binding affinity and localized signaling.

The ErbB family of four closely related receptor tyrosine kinases (RTKs) – the epidermal growth factor receptor (ERBB1/EGFR), ERBB2/HER2, ERBB3/HER3, and ERBB4/HER4 – is implicated in various invasive diseases for promoting aberrant cell survival, proliferation, and migration. Multiple antibodies and kinase inhibitors have been clinically approved for targeting ErbB-family signaling in oncology, including the epidermal growth factor receptor (EGFR) blocking antibody cetuximab. Dysregulated ErbB signaling can occur in a ligand-independent manner, for example via receptor mutation or amplification, and also in a ligand-dependent manner where co-expression of both the receptor and its ligand allows cells to signal to themselves in an autocrine process. As evidence for the latter, responsiveness to EGFR inhibitors correlates with expression of its cognate ligands such as amphiregulin (AREG), generally in patients with wildtype EGFR^1^,^2^. Despite some clinical success, EGFR and HER2 inhibitors invariably lose efficacy as cancers develop resistance, often arising from enhanced ligand-dependent ErbB signaling. ErbB family receptors can be activated by 11 known ligands that activate subsets of the 4 ErbB receptors with varying degrees of affinity. Frequently, inhibition of a single ErbB family member becomes ineffective due to bypass signaling through alternative receptors^3^; for example, upregulation of the ERBB3 and ERBB4 ligand heregulin mediates cetuximab resistance^4^. In some cases, EGFR inhibition can be outcompeted by upregulation of certain high affinity ligands such as transforming growth factor alpha (TGFα)^5^. These two effects are combined in the case of heparin-binding epidermal growth factor (HB-EGF), which activates both EGFR and ERBB4 at high affinity and similarly leads to cetuximab resistance^6^. This evidence has ultimately motivated the development of complimentary strategies for targeting ErbB-family signaling that extends beyond direct binding and inhibition of EGFR and HER2.

Inhibiting ErbB-ligands themselves, rather than their receptors, represents one promising alternative strategy to target ErbB-family signaling. Because many ErbB ligands (including AREG, TGFα, and HB-EGF) are proteolytically shed from the cell-surface, several implicated proteases have become attractive drug targets. In particular, A Disintegrin and Metalloproteinase 10 and 17 (ADAM10 and ADAM17) have been targeted for their role in shedding ErbB-family ligands^7^. However, most small molecule metalloproteinase inhibitors exhibit poor specificity and have largely failed in the clinic due to serious toxicological issues. Although more specific ADAM10 and ADAM17 inhibitors have recently been developed^8^,^9^,^10^, these proteases may in fact be problematic as drug targets owing to their promiscuous substrate preferences^11^,^12^,^13^. To specifically target ErbB ligands themselves, Fc fusion proteins of ErbB receptors and so-called “decoy” antibodies that complex with ligands and prevent them from binding cell-surface receptors have been developed. However, these approaches often fail to substantially reduce growth in tumors that were known to be responsive to traditional anti-ErbB therapies^14^,^15^, and the mechanisms for their failure remain unclear. Consequently, a need exists to better understand why these decoy-Ab approaches have not been more successful and to identify improved and complimentary strategies for inhibiting ErbB signaling activity.

Here, we hypothesized that systems-level computational modeling of autocrine signaling would provide a quantitative understanding of how multiple ErbB-family ligands contribute to overall cell behavior, and how biochemical differences among ligands may influence corresponding therapeutic strategies to target them. We focused on ErbB-dependent cell-migration in a model of endometriosis, which is a disease characterized by the presence of endometrial-like tissue outside of the uterus, most commonly in the form of invasive peritoneal lesions and ovarian endometriomas. Based on computational results and validated by experimental tests, we found that a decoy antibody ineffectively blocked HB-EGF compared to AREG, due to the high affinity and consequently localized autocrine signaling behavior of HB-EGF. As an alternative strategy, we inhibited HB-EGF activity by targeting its cleavage from the cell surface. We found that ADAM12 activity correlated closely with HB-EGF shedding in endometriosis; therefore, we developed a specific inhibitor of ADAM12 based on its recombinant prodomain (PA12) to reduce HB-EGF shedding, and demonstrated it as effective. Taken together, these results i) provide a quantitative explanation of limiting factors in using decoy antibodies against growth-factor ligands, particularly relevant to high affinity ligands such as HB-EGF; ii) demonstrate ADAM12 as a relevant sheddase of HB-EGF in endometriosis; and iii) present a novel, specific ADAM12 inhibitor to reduce HB-EGF shedding and resulting cell migration behavior.

## Results

### A computational model of autocrine signaling accurately predicts that HB-EGF signaling is more localized than the low-affinity ligand, amphiregulin (AREG)

**Figure 1:**
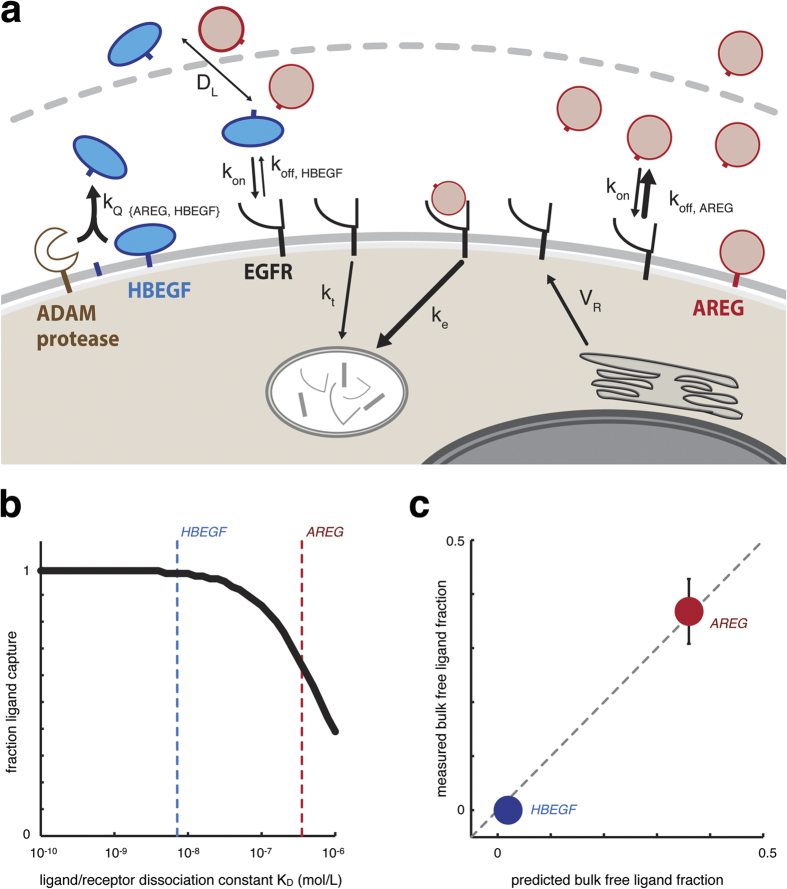
Computational model of autocrine signaling accurately predicts ligand capture differences between AREG and HB-EGF. (**a**) Schematic of computational model that incorporates receptor trafficking combined with the proteolytic release, spatial diffusion, and receptor-mediated capture of growth factor ligands. (**b**) Ligand capture depends on ligand-receptor binding affinity, computationally modeled and represented here as the cumulative fraction of proteolytically released ligand that is captured by EGFR over the course of 24 h. (**c**) The computational model accurately predicts that HB-EGF is captured at higher levels compared to AREG. The fraction of bulk free ligand, equivalent to (1 - [*fraction ligand capture*]), was experimentally measured by taking the ratio of supernatant ligand concentrations after 24 h with or without the EGFR blocking antibody, mAb225 (n = 2 ± SEM).

To study how varied physicochemical properties of ErbB-ligands influence overall autocrine signaling behavior, we developed a model of EGFR signaling based on ordinary differential equations (ODEs) that described receptor production and internalization, ligand shedding and localized diffusion, and ligand-receptor binding (Fig. 1A). The model was based on previous computational implementations^16^,^17^ along with evidence for ErbB-ligand release primarily through proteolysis^13^ (Fig. S1A), and was modified here to explicitly match quantitative experimental measurements in a tissue culture model of endometriosis (see *Methods*; Table S1,2). For simplicity, the model assumes endocytosis via ErbB receptor binding and does not explicitly account for alternative interactions with tetraspanins, integrins, extracellular matrix factors, and secretion pathways. Nonetheless, lumped modeling parameters capture many of these features’ effects implicitly, and their implications are further discussed elsewhere in the manuscript. This work used 12Z, a commonly studied immortalized cell line that was isolated from an endometriotic biopsy^18^. EGFR is highly over-expressed in these cells, and among several ErbB-ligands (including EGF, TGFα, NRG1b, and betacellulin) we found AREG and HB-EGF to be the most highly expressed (Table S1). Interestingly, HB-EGF and AREG exhibit distinct physicochemical properties, with more than an order of magnitude difference in binding affinity to EGFR^19^. We hypothesized that the greater HB-EGF binding affinity would cause more localized signaling, and therefore probed the computational model to test this hypothesis. We systematically varied the ligand/receptor dissociation constant K_D_ in the model and calculated the cumulative fraction of released ligand that was subsequently re-captured by surface receptors over the course of 24 h. This analysis showed that binding affinity substantially impacted ligand capture: ligands with dissociation constants below 10 nM were nearly entirely captured, while <40% of ligand was captured from low-affinity ligands with dissociation constants above 1 μM (Fig. 1B). To experimentally confirm this observation, we treated 12Z with a non-humanized version of cetuximab (mAb225) to block ligands from binding EGFR. Comparison of ligand accumulation ± mAb225 enabled direct calculation of the fraction of free, uncaptured ligand, and these fractions were dramatically different between HB-EGF and AREG. The high-affinity HB-EGF was nearly completely re-captured by cells, while the low-affinity AREG was able to diffuse away and accumulate in the bulk supernatant (Fig. 1C). Importantly, these results accurately matched predictions from the computational model that were based solely on differences in ligand binding affinity. To test the generalizability of the model, we examined autocrine ligand capture across a panel of cancer cell lines and found similar behavior: while low-affinity AREG was detectable in bulk supernatant, nearly all HB-EGF and the similarly high-affinity ligand TGFα (transforming growth factor α) were re-captured by surface receptors (Fig. S1B,C). Overall, these results demonstrate that HB-EGF and AREG exhibit distinct degrees of signaling localization, likely owing to the direct impact of ligand/receptor binding affinity on the proportion of ligand that escapes capture.

### Efficacy of decoy antibodies depends on ligand properties and the degree of signaling localization

**Figure 2:**
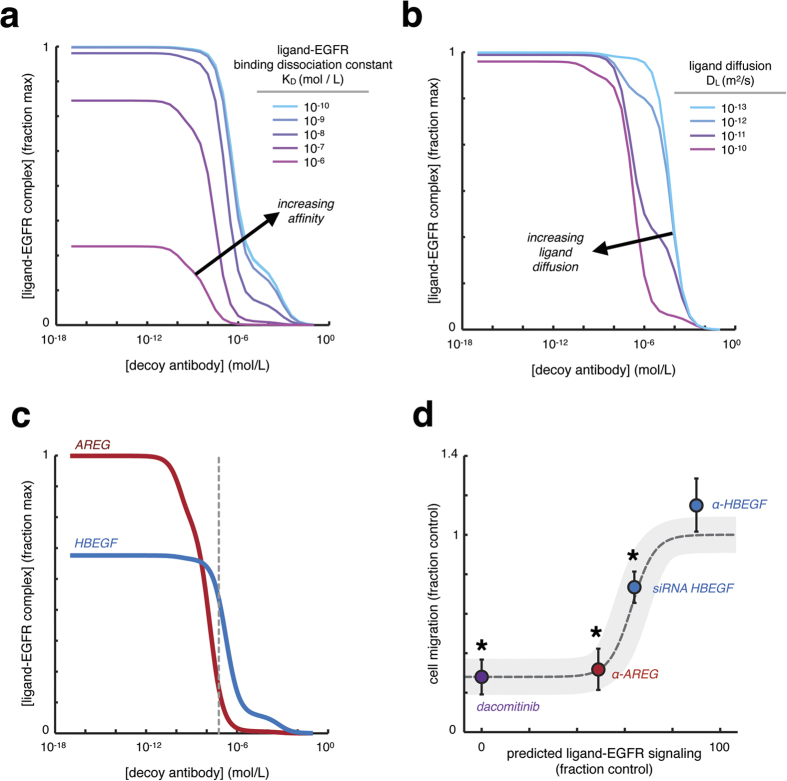
Ligand properties significantly influence decoy antibody efficacy. (**a**,**b**) Computational modeling shows that increasing ligand binding affinity (**a**) or decreasing ligand diffusion (**b**) substantially reduce the ability of decoy antibodies to effectively sequester ligands and prevent them from binding to surface EGFR. Modeling results were obtained after simulating 24 h decoy antibody treatment. (**c**) HB-EGF and AREG exhibit differential sensitivity to decoy antibodies, based on computational modeling that incorporated measured ligand release and receptor levels in 12Z endometriotic cells, along with known differences in HB-EGF and AREG binding affinity to EGFR. Dashed line marks 10 μg/mL, the experimentally tested concentration of decoy antibodies. (**d**) Using 12Z, computational estimates of ligand-EGFR complex levels at 24 h post-treatment correlate with cellular migration observed over the course of 24 h in collagen I gels. Note neither the model nor the cell migration measurements show HB-EGF decoy antibody (α-HBEGF) to have a significant effect, in contrast to the AREG decoy antibody (α-AREG). Genetic HB-EGF knockdown confirms a role for HB-EGF in cell migration, and complete inhibition of ErbB signaling using dacomitinib serves as a positive control (n ≥ 3 ± SEM; *p < 0.05, two-tailed t-test, reduction in cell migration).

We next hypothesized that the extent of signaling localization may have significant therapeutic consequences, especially for decoy antibodies that have been developed to sequester growth factors and cytokines and thus block their activity. To examine this, we expanded the computational model to include a soluble decoy antibody capable of reversibly binding to ligand at sub-nanomolar affinity (K_D_ = 0.1 nM), consequently blocking ligand interaction with surface growth-factor receptors. We then studied how decoy antibody treatment affected autocrine signaling in a dose-dependent manner, and how this relationship depends on relevant ligand properties. Because we previously found that ligand binding affinity impacts signaling localization, we used the computational model to test the impact of binding affinity on decoy antibody efficacy. Indeed, this analysis suggested that increased binding affinity of the ligand to its cognate receptor confers resistance to decoy antibody treatment (Fig. 2A). ErbB-family ligands exhibit variable rates of effective diffusion; for instance, transport of HB-EGF can be significantly limited by direct binding to extracellular matrix^20^ or surface-associated proteoglycans such as heparin-sulfate proteoglycan (HSPG)^21^. We used the computational model to test the impact of effective ligand diffusion on decoy antibody behavior, and found that decreased diffusion limited therapeutic efficacy (Fig. 2B). We next used the computational model to compare decoy antibody sensitivity between AREG and HB-EGF in the 12Z endometriosis cell line. Consistent with results above, the model suggested that the AREG was more sensitive to decoy antibody treatment compared to HB-EGF (Fig. 2C). Furthermore, this comparison did not consider differences in ligand diffusion, which would only enhance the differential antibody sensitivity. Of note, these modeling results describe generic release of ligands from the cell surface, and therefore do not explicitly depend on the mechanism of ligand production (whether through secretion, proteolysis, or other pathways). Overall, these modeling results suggest that ErbB ligands may exhibit distinct sensitivity to decoy antibodies, largely because of their differentially localized signaling.

### α-AREG but not α-HBEGF decoy antibody reduces endometriotic cell migration

Based on the above computational predictions, we next compared the ability of α-AREG and α-HBEGF decoy antibodies to reduce cellular migration in endometriotic tissue culture. 12Z cell migration strongly depends on constitutive autocrine EGFR signaling^13^. Treatment with exogenous recombinant HB-EGF stimulates motility in 12Z, while blocking basal autocrine EGFR signaling using mAb225 reduces motility (Fig. S2A,B^13^). Furthermore, treatment with a pan-ErbB kinase inhibitor (dacomitinib) dramatically reduces cell migration (Fig. S2C), even at concentrations that didn’t significantly impact cell growth (Fig. S3). We treated 12Z with either α-AREG (see^13^) or α-HBEGF decoy antibodies at a relatively high concentration (10 μg/mL; dashed line, Fig. 2C), and measured cell migration over 24 h. In agreement with the computational model’s prediction, α-AREG was effective in reducing cell migration, while α-HBEGF was not (Fig. 2D). To confirm that endogenously expressed HB-EGF contributes to cell migration, we genetically silenced HB-EGF with siRNA. This treatment reduced migration by approximately 30% (Fig. 2D; see Fig. S4A for siRNA knockdown efficiency), which was roughly commensurate with the computational model’s predicted 35% decrease in total receptor signaling (Fig. S4B). To verify that the decoy antibody was functional in the absence of highly localized signaling, we administered exogenous recombinant HB-EGF 30 min following α-HBEGF antibody treatment. The computational model predicted that exogenous HB-EGF would be more efficiently blocked by decoy antibody treatment compared to endogenous HB-EGF (Fig. S5A), largely owing to the former’s non-localized nature. The experimental data indeed confirmed this prediction (Fig. S5B). Overall, these results underscore how the degree of signaling localization significantly impacts decoy antibody efficacy, and highlights how this localization differs between AREG and HB-EGF as two model ErbB ligands.

### Cue-signal-response analysis of endometriosis cell culture suggests a significant role of ADAM12 in HB-EGF shedding

**Figure 3:**
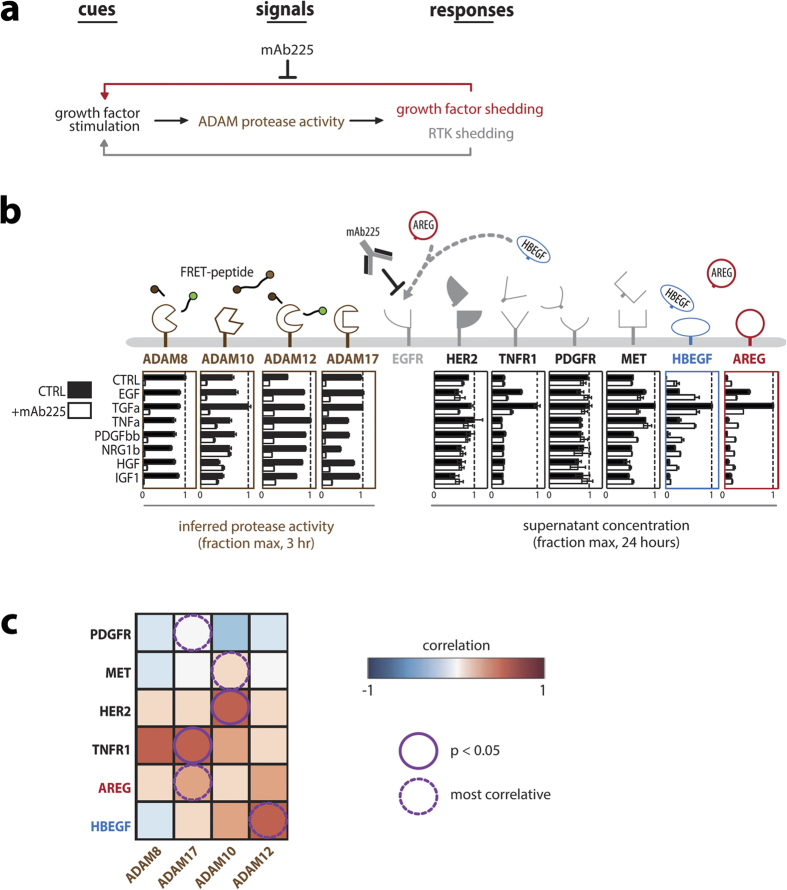
Cue-signal-response data set relates protease activities to ectodomain shedding in endometriosis cell culture. (**a**) Overview schematic of cue-signal-response modeling approach to infer biochemical relationships between exogenously applied signaling cues, measured ADAM proteolytic activity “signals,” and corresponding ADAM-substrate shedding “responses.” (**b**) Serum-starved 12Z cells were treated with growth factor/cytokine “cues” for 3 h and simultaneously monitored in real-time for live-cell protease activity “signals” using FRET-based polypeptide probes. *Left:* Specific ADAM activities were then computationally inferred using the PrAMA algorithm (n = 4 ± SEM). *Right:* 24 h later, supernatants were analyzed using ELISA for endogenous ADAM-substrate accumulation “responses” (n = 3 reps ± SEM). (**c**) Pairwise Pearson correlations between protease activities ((**b**), left) and ADAM-substrate shedding ((**b**), right) were calculated for measurements as they varied across the panel of growth-factor/cytokine treatment conditions; results describe correlational relationships between patterns of inferred ADAM catalytic activity and downstream substrate proteolysis. EGF-ligand correlations were performed only for the n = 8 growth-factor treatment conditions in the presence of mAb225 to block ligand endocytosis, while receptor correlations were calculated with and without mAb225 (n = 16 conditions from n ≥ 3 reps; p-value from two-tailed t-tests).

Considering the above challenges in using decoy antibodies to block localized HB-EGF signaling, we next turned to specific protease inhibition as an alterative therapeutic strategy. In addition to blocking generation of soluble HB-EGF ectodomain in the cell supernatant, protease inhibition may also prevent the generation of c-terminal HB-EGF fragments that traffic to the nuclear membrane and influence transcriptional activity^22^,^23^. Indeed, using immunofluorescence we found that α-HBEGF decoy antibodies had no significant impact on nuclear accumulation of HB-EGF c-terminus, while metalloproteinase inhibition did (Fig. S6). We next examined which particular metalloproteinases mediate HB-EGF shedding in endometriotic cells. Although dysregulated ADAM proteolytic activity, ectodomain shedding, and EGFR autocrine signaling are clinically associated with endometriosis^13^,^24^,^25^, little functional data exists that specifically describe proteases implicated in HB-EGF shedding for the disease. Ectodomain shedding and ADAM proteolytic activity comprise a complex network of overlapping specificities and context-dependent biochemical interactions. Therefore, we initially undertook a “cue-signal-response” (CSR) computational analysis to delineate the principal contribution of different ADAM proteases to HB-EGF ectodomain shedding (Fig. 3A). By stimulating cells with disease-relevant environmental “cues”, monitoring specific ADAM proteolytic activity “signals,” and comparing them with corresponding measurements of ectodomain shedding “responses,” the CSR modeling paradigm enabled inference of biochemical interactions in a systematic, non-invasive, and comprehensive manner. As cues in this study, we treated cells with exogenous growth factor ligands and the inflammatory cytokine tumor necrosis factor alpha (TNFa), which have all been previously implicated in disease^13^. Across this set of growth factor treatment “cues,” we recorded the ADAM proteolytic activity “signals” over the first 3 h of stimulation using Protease Activity Matrix Analysis (PrAMA), which is a combined experimental/computational approach that relies upon panels of soluble FRET-based polypeptide protease substrates to ascertain the relative catalytic activity of specific metalloproteinases in live cells^26^. Finally, we correlated protease activity measurements with accumulation of endogenous ADAM substrates in cellular supernatant (Fig. 3B). To prevent EGFR uptake of endogenously release EGF-ligands, which could confound the correlational analysis, we furthermore performed the experiments in the presence of mAb225.

We compared the protease activity “signals” with ectodomain cleavage “responses” to identify which ADAM activities most correlated with HB-EGF accumulation. Results suggested that ADAM12 plays a central role in HB-EGF shedding in endometriotic cell culture: ADAM12 activity most strongly correlates with HB-EGF supernatant accumulation, and vice-versa (Fig. 3C). As encouraging validation, this analysis identified HER2 and MET receptor shedding as most closely correlating with ADAM10, which is thought to be their principal sheddase. Furthermore, ADAM17 was most associated with its well known substrate, TNFa receptor 1 (TNFR1). ADAM8 activity was also associated with TNFR1 release, and ADAM8 cleavage of TNFR1 has been previously observed in the context of neurodegeneration^27^. Thus, the computational CSR analysis here provides evidence that ADAM12 mediates HB-EGF shedding in endometriosis, and therefore represents a promising avenue of investigation.

### Recombinant PA12 can be synthesized in E. coli and is a specific inhibitor of ADAM12

**Figure 4:**
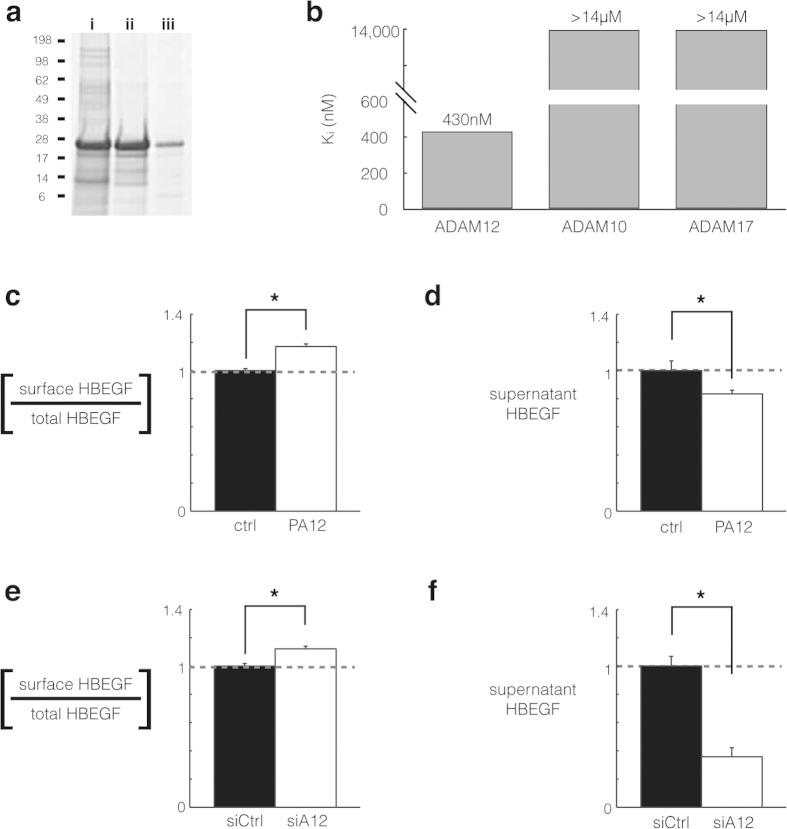
ADAM12 inhibition using ADAM12 prodomain (PA12) reduces HB-EGF shedding in endometriosis cell culture. (**a**) Coomassie-stained SDS-PAGE gel showing PA12 isolation from E. coli inclusion bodies (i); its purification from Ni-NTA affinity chromatography (ii); and its subsequent refolding, dialysis, and concentration (iii). (**b**) PA12 inhibits recombinant ADAM12 but not recombinant ADAM-10 or -17, measured by inferring inhibitory constants (K_i_) from dose-response curves in a fluorogenic FRET-peptide based assay. (n = 2 ± SD). (**c,d**) 2 μM PA12 treatment for 3 h increases relative levels of full-length HB-EGF on the cell surface. 12Z-HE cells stably expressing HB-EGF with Myc-tagged ectodomain and a GFP-tagged cytoplasmic tail were stained, fixed, and analyzed by flow-cytometry. (**d**) Corresponding to *c*, 3 h PA12 treatment reduces supernatant accumulation of HB-EGF, measured by ELISA. (**e,f**) Genetic ADAM12 knockdown increases relative levels of full-length HB-EGF on the cell surface (**e**), while decreasing its accumulation in the supernatant (**f**). 12Z-HE cells were treated with siRNA for 72 h, supernatant was exchanged, and 3 h later cells were analyzed by flow cytometry (**e**) and supernatant HB-EGF was measured by ELISA. (**c–f**) (n ≥ 3 ± SEM); *p < 0.05; two-tailed student’s t-test.

We postulated that an isolated prodomain of ADAM12, as was found for ADAM9^28^ and ADAM10^8^, could be used here as a specific inhibitor to block ADAM12-mediated ectodomain shedding events. The mammalian-expressed prodomain of ADAM12 associates with ADAM12 in serum, and co-purifies with the proteinase from mammalian cells^29^,^30^. Therefore we hypothesized that the prodomain could be efficiently expressed in E. coli and serve as an effective, specific ADAM12 inhibitor. We expressed His-tagged human ADAM12 prodomain (amino acids 29–206), isolated it from inclusion bodies, purified it using a Ni-NTA resin, and prepared it by concentration and dialysis (Fig. 4A). This final ADAM12 prodomain product (PA12) was then tested for inhibitor potency and specificity using an *in vitro* fluorogenic protease activity assay and purified recombinant ADAM-10, -12, and -17. The inhibitory constant ***K***_***i***_ for ADAM12 was 430 ± 110 nM, and no detectable inhibition was observed for either ADAM10 or ADAM17 (Fig. 4B). Overall, these results suggest that PA12 can be recombinantly expressed, isolated, and used to inhibit purified ADAM12 in a specific manner.

### ADAM12 inhibition reduces HB-EGF ectodomain shedding and cell migration

We next investigated the ability of PA12 to reduce HB-EGF shedding in endometriosis cell culture. To enable accurate single-cell quantification of HB-EGF cleavage, we monitored ectodomain shedding using a transgenic HB-EGF construct in 12Z (12Z-HE cells) that allows for simultaneous measurement of total expression via GFP fused to the cytoplasmic tail, and a Myc epitope-tagged extracellular domain for determination of intact HB-EGF on the cell surface. Live-cell immunostaining of the Myc epitope combined with flow cytometry showed that PA12 treatment for 3 h increased the relative level of intact transmembrane HB-EGF in 12Z by nearly 20% (Fig. 4C; Fig. S7A,B). Supernatants from 12Z-HE cells were assessed by ELISA and indicated a similar decrease in HB-EGF accumulation after 3 h of PA12 treatment (Fig. 4D). The combined increase in intact HB-EGF on the cell surface and decrease in soluble HB-EGF in the cell supernatant strongly suggest PA12 treatment reduces HB-EGF ectodomain shedding.

We compared changes in HB-EGF shedding from PA12 treatment with shedding changes in response to genetic ADAM12 silencing. We used siRNA treatment to knockdown ADAM12 (see Fig. S8 for knockdown efficiency) and again used the 12Z-HE transgenic HB-EGF reporter system to monitor HB-EGF. Comparable to the effects of PA12, ADAM12 knockdown yielded a 10–15% increase in the relative level of intact transmembrane HB-EGF in 12Z-HE (Fig. 4E). Supernatant HB-EGF correspondingly decreased by about 60% (Fig. 4F). The longer-term siRNA treatment slightly decreased total HB-EGF expression levels by 14% (Fig. S7C,D), which may explain why supernatant levels changed more dramatically than relative surface levels in this case. Nevertheless, the combined increase in intact HB-EGF on the cell surface and decrease in soluble HB-EGF in the cell supernatant indicate that ADAM12 genetic knockdown reduces HB-EGF ectodomain shedding, similar to as observed with PA12 treatment. To examine the generalizability of these results, we also used siRNA to silence ADAM12 in a panel of cancer cell lines. In agreement with the 12Z results, knockdown in ADAM12 expression reduced supernatant HB-EGF accumulation in 3/4 of the tested cell lines (Fig. S9). Of note, ADAM12 siRNA reduced HB-EGF supernatant accumulation more significantly than ADAM10- or ADAM17- targeted siRNA, which did not affect HB-EGF shedding under basal conditions in the same 12Z cell line tested here^13^, thus suggesting ADAM12 is a primary HB-EGF sheddase. Overall, these results provide confirmatory evidence that ADAM12 contributes to HB-EGF shedding, and that PA12 has the capability to modify cell-surface proteolysis.

**Figure 5:**
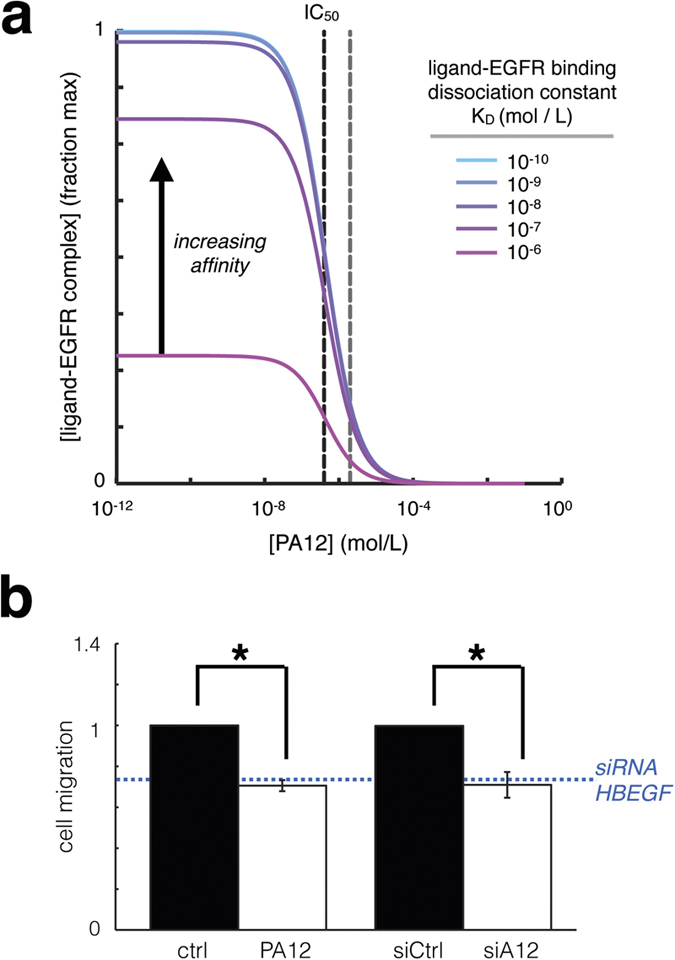
Protease inhibition blocks autocrine signaling independent of ligand-receptor binding affinity, and ADAM12 inhibition reduces endometriotic cellular migration. (**a**) In contrast to decoy antibody efficacy, the predicted ability of PA12 to block HB-EGF autocrine signaling does not depend on ligand-receptor binding affinity. Gray dashed line at right indicates the used PA12 concentration (2 μM). (**b**) PA12 treatment and genetic ADAM12 knockdown significantly reduce 12Z cell migration in collagen I gels, measured over 24 h of treatment, compared to their respective controls (*n = 3 ± SEM; p < 0.05, two-tailed student’s t-test).

Given ADAM12 inhibition reduces HB-EGF shedding, we next examined if it would likewise reduce endometriotic cell migration. In contrast to decoy-antibody strategies, computational modeling suggested that the effectiveness of protease inhibitors in limiting ligand-mediated autocrine signaling does not depend on the ligand properties such as ligand-receptor binding affinity (Fig. 5A). In fact, the amount of protease inhibitor required to block 50% of ligand-receptor complex (IC_50_) was modeled to be completely independent of binding affinity. In agreement with this modeling prediction, we found that ADAM12 inhibition using either PA12 or genetic ADAM12 knockdown reduced cell migration to a similar degree compared to genetic HB-EGF knockdown (Fig. 5B). Given multiple ErbB-ligands (including AREG) and proteases (including ADAM10) also drive cell migration in these cells^13^, it is unsurprising that cell migration was not completely blocked. However, these results nevertheless demonstrate that ADAM12 inhibition not only reduces HB-EGF shedding, but also represents a complementary therapeutic strategy to diminish cell migration in endometriosis.

## Discussion

Here, we used computational modeling of ErbB-family signaling to study how growth factor properties such as ligand binding affinity impact the degree of autocrine signaling localization. Although previous studies have used experimental and computational approaches to analyze EGFR autocrine signaling^16^,^17^,^31^,^32^,^33^,^34^ and ligand-receptor binding constants^35^, in general the differences between individual ErbB-family ligands and their resulting therapeutic implications have not been fully appreciated. We integrated precise measurements of endogenous autocrine signaling with available biophysical parameters to develop a quantitative model that could predict distinct behavior between two prominent growth factors, AREG and HB-EGF. These studies ultimately provided accurate explanations for why decoy antibodies targeting AREG were effective, while antibodies targeting HB-EGF were not in this context. In essence, we found that HB-EGF exhibited highly localized autocrine signaling behavior owing largely to its high binding affinity compared with AREG. This localization significantly prevented decoy antibodies from effectively sequestering ligand. Previous studies have highlighted the spatially restricted nature of HB-EGF signaling *in vivo*, and evidence suggests that localization may be even further enhanced in several disease contexts through cell-cell contact, binding to extracellular matrix and proteoglycans, mechanical tissue compression, and diffusion-limiting barriers such as the basement membrane^36^,^37^,^38^. While both HB-EGF and AREG are implicated in a wide range of diseases, AREG has been less associated with highly localized signaling compared to HB-EGF. For instance, compared to other growth factors AREG has been more associated with signaling across cell-types such as tumor-associated macrophages^39^ and fibroblasts^40^,^41^,^42^. Overall, this work highlights how different ligand properties may govern spatially distinct behavior with implications for both understanding biological mechanisms and in developing effective therapies.

Based on the results showing highly localized HB-EGF signaling, we turned towards specific protease inhibition as an alternative strategy to block signaling activity. Multiple ADAM proteases have been implicated in shedding HB-EGF from the cell surface^43^, yet protease-ligand processing occurs in a highly context-dependent and cell-type dependent manner^13^. Therefore we performed a correlative multivariate analysis to identify the primary ADAM protease responsible for HB-EGF shedding in endometriosis, and found ADAM12 to be most associated. Indeed, ADAM12 has been previously implicated in HB-EGF shedding^44^,^45^,^47^, and ADAM12 represents a compelling drug target through clinical association with diseases including hypertension, asthma, liver fibrosis, obesity, adverse pregnancy outcome, along with multiple cancers^48^. Most relevant to the endometriosis study here, large-scale genetic linkage studies^49^ and gene-expression analyses^50^ implicate ADAM12 in endometriosis development, and clinical studies additionally show evidence of dysregulated HB-EGF^51^ with disease. In addition to these correlations, genetic ADAM12 overexpression or silencing causes compelling phenotypic changes in animal models of both cancer and obesity^44^,^53^,^54^,^55^. Furthermore, metalloproteinase inhibition primarily targeting ADAM12 protects mice from cardiac hypertrophy^45^. These studies highlight ADAM12’s importance not just in the context of HB-EGF signaling in endometriosis, but for a wide range of pathologies. Consequently, a specific and potent inhibitor of ADAM12 may have potential as both a therapeutic and as a tool for studying the role of ADAM12 in pathological and physiological processes alike.

Here we report recombinant PA12 as a specific inhibitor of ADAM12 with sub-micromolar potency, and demonstrate its application to reduce HB-EGF shedding and cellular migration in endometriosis cell culture. By exhibiting selectivity over closely the related ADAM10 and ADAM17 proteases, we anticipate that PA12 will be a useful tool in parsing functional differences between ADAM family enzymes. Moreover, PA12 has the potential for therapeutic application to the many diseases involving ADAM12 dysregulation, especially if the prodomain can be further engineered to achieve greater potency and solubility. We found that ADAM12 inhibition via both PA12 treatment and genetic knockdown decreased HB-EGF shedding in endometriosis cell culture, while additionally reducing cell migration. In sum, these results show PA12 as an inhibitor of ADAM12 capable of modulating cellular shedding events, and further provide evidence that HB-EGF shedding contributes to the migratory behavior of endometriotic cells. Future work is needed to understand the functional implications of ADAM12 inhibition in animal models of endometriosis along with other models of disease, and PA12 represents a promising tool for that aim.

Over the past 10 years, inhibitors have been made to several of the ADAM family of proteinases, most prominently ADAM10 and ADAM17. Unfortunately, most small molecule agents exhibit poor specificity and have often failed in clinical trials due to serious toxicological issues^7^,^56^,^57^. Important distinctions exist between related ADAM family proteinases, including ADAM10, ADAM17, and ADAM12, and evidence suggests that ADAM10 may in fact be an anti-drug target in several contexts. For example, in the context of invasive diseases like cancer, ADAM10-mediated MET shedding can attenuate downstream pro-survival, pro-growth, and pro-metastatic signaling^11^,^12^. In contrast, ADAM12 represents a promising target for its role in HB-EGF shedding, and yet is not widely associated with concomitant shedding of receptor tyrosine kinases or amyloid peptides^48^. Compared to small-molecules, biologics have become a promising avenue for developing high selectivity. The prodomains of ADAM9 and ADAM10 selectively inhibit their targets and have been successfully utilized in a variety of applications^8^,^13^,^24^,^28^,^58^, and PA12 presented here exhibits comparable specificity and sub-micromolar potency. Antibodies^9^,^10^,^59^,^60^ and genetically engineered tissue inhibitor of metalloproteinases (TIMPs)^61^ have also been used to specifically inhibit metalloproteinases. In sum, these advances combined with the PA12 developed here highlight the potential of biologics as therapeutics and tools for elucidating the physiological substrates, roles and mechanisms of the enzymes they target.

Overall, here we have demonstrated i) that differences in ligand-receptor affinity can lead to distinct behavior among closely related ErbB ligands, with significant therapeutic consequences; ii) that ADAM12 proteolytic inhibition represents an attractive alternative to block even highly localized HB-EGF signaling; and iii) that recombinant ADAM12 prodomain can be used as an effective, specific inhibitor of ADAM12. We anticipate that these conclusions will readily extend to diseases and biological processes beyond endometriosis; that the quantitative principals of localized signaling may be applied to additional extracellular signaling systems; and that recombinant PA12 may be applied or further refined as a therapeutic or tool compound.

## Methods

### Cell Culture Materials & Methods

The 12Z cell line was generously provided by Anna Starzinski-Powitz (University of Frankfurt) by way of Steve Palmer (EMD Serono). Cell line authenticity was determined by short tandem repeat (STR) profiling (GRCF DNA services; Johns Hopkins University) following the ANSI/ATCC ASN-0002-2011 Authentication of Human Cell Lines standardized procedure. An 80% match threshold from 8 STR loci was not met with any other cell type using either the ATCC (Masters algorithm) or ANSI (ANSI algorithm) methods when compared against cell line databases, of which 12Z was not a member. Moreover, previous matrix metalloproteinase (MMP) profiling^13^ of MMP-1, -2, -3, -7, and -9 matched levels reported elsewhere for 12Z, as did profiling of TIMPs and EGFR^62^. 12Z were routinely cultured in media that consisted of DMEM/F12 supplemented with 100 U/ml penicillin, 100 μg/ml streptomycin (Invitrogen), along with 10% fetal bovine serum (Atlanta Biologicals; Atlanta, GA) at 37^o^ C, 5% CO_2_. Cancer cell lines were obtained from ATCC and Asterand (SUM149PT) and were cultured according to manufacturer’s guidelines. siRNA treatments used ON-TARGETplus SMARTpool siRNA (Thermo Scientific), with siGENOME non-targeting siRNA pool-2 as the negative control. 0.5 million cells were seeded in 10 cm dishes. The following day the cells were transfected using 5 μL Dharmafect 4 and 125 pmol siRNA according to the manufacturer’s protocol. One day after transfection, cells were reseeded for knockdown experiments; 48 h after transfection cells were analyzed for cell migration or ligand shedding; 72 h after transfection cells were analyzed for protease activity and lysed for knockdown validation. ADAM12 siRNA knockdown efficiency was validated by western blot using manufacturer guidelines (Nu-PAGE SDS-PAGE system; Life Tech.) with biotinylated anti-ADAM12 antibody (R&D Systems), Strep-680 near-infrared labeling, and Licor Odyssey gel imaging. ADAM12 knockdown in cancer cells and HB-EGF knockdown efficiency was validated by ELISA using manufacturer’s guidelines (R&D systems), with lysate normalized by protein content (measured by microBCA assay; Pierce). Recombinant HB-EGF (Peprotech; Rocky Hill, NJ), dacominitib (Selleck Chem); mAb225 (EGFR blocking mAb; 10 μg/ml; purified from the ATCC hybridoma); AREG and HB-EGF decoy antibodies (R&D systems) were purchased from commercial vendors.

### Immunoassays

Live-cell immunostaining and flow cytometry were used to assess surface levels of HB-EGF and EGFR. For HB-EGF staining, stably transduced 12Z Myc-HBEGF-GFP cells^13^ were serum starved for 4 h and treated with fresh serum-free media for 3 h in the presence of PA12 or control buffer. Cells were treated with trypsin/EDTA (Gibco) for 15 min., rinsed in 4 °C PBS + 3% FBS, and incubated with primary anti-Myc antibody at 1:100 dilution for 2 h. in 4 °C PBS + 3% FBS. Cells were rinsed in 4 °C PBS +   3% FBS, fixed in 2% PFA + PBS overnight, and stained with secondary antibody conjugated to Alexa647 the following day. Flow cytometry was performed using the BD Biosciences LSR-II. Supernatant HB-EGF concentration from these experiments was measured by ELISA using manufacturer’s guidelines (R&D Systems). EGFR surface staining was performed as described previously^63^, using the α-EGFR antibody mAb225 at 10 μg/ml and using a 5-point standard curve based on antibody-coated beads (Bangs Labs; Fishers, IN) to deduce absolute receptor numbers (***R***).

For dual immunostaining of N-terminal and C-terminal HB-EGF, 12Z were plated O/N in 96-well plates (Ibidi), treated for 24 h, washed 3× in PBS, and immediately fixed in 4% PFA at room temperature for 30 min. Cells were permeabilized with 0.1% Triton × 100 for 5 min, washed, and blocked O/N in Odyssey Blocking Buffer (Li-cor) at 4 °C. Cells were incubated O/N with primary antibodies for the C-terminus (C-18; Santa Cruz) or N-terminus (clone 406316; R&D Systems) at 4 °C, washed, incubated with Alexa488- or Alexa647- conjugated secondary antibodies (Invitrogen) O/N, washed, and were imaged on an inverted A1R laser scanning confocal microscope (Nikon).

### Endogenous ligand shedding

Cells were plated in 12-well plates overnight at 1.5 million cells per well in 500 μl full serum media. The following day, they were pre-treated with either 10 μg/ml mAb225 or 10 μM BB94 for 30 min, followed by treatment with 1 μM PMA. 24 h later, media was collected, clarified by centrifugation (300 × g, 5 min), and frozen for subsequent ELISA analysis (Duo-set; R&D Systems). Cells were immediately trypsinized and analyzed for cell count and viability using ViCell (Beckman Coulter), which was then used for supernatant normalization. Basal and PMA-stimulated ligand release rates (***k***_***Q***_***P***) were measured in the presence of mAb225. These values, along with ligand capture ratios (determined by ligand +/– glyph mAb225), were calculated after background subtraction using the BB94-treated samples as a negative control. For deriving absolute quantities in the ELISA, freshly reconstituted recombinant ligand was used for a >5-point standard curve per manufacturer’s instructions. Precise protease levels (***P***) did not significantly impact the overall model results within realistic ranges (500–50,000 per cell) and were estimated at 5000 per cell. Initial screening for AREG, HB-EGF, EGF, and betacellulin were performed as described above but using multiplexed bead-based immunoassay for Luminex (Millipore), according to manufacturer’s guidelines. HB-EGF and AREG levels were measured as >4-fold higher than EGF and betacellulin levels in 12Z. TGFα was measured in cancer cell lines by ELISA (Duo-set; R&D Systems).

### Cell migration assays

For endpoint cell migration assays, 12Z were mixed with DMEM +2.2 mg/ml rat tail collagen I (BD Biosciences) on ice, placed in a standard 96-well tissue culture plate at 50 μl and 5000 cells per well, spun for 5 min at 300 × g, and allowed to polymerize at 37^o^ C. 50 μl full serum media containing inhibitors, growth factors (added 30 min following inhibitors when appropriate) or the relevant buffer control was then added to the wells, and the plate was incubated for 24 h. Gels were then fixed with 1% paraformaldehyde, stained with YO-PRO-1 (Invitrogen), and imaged with a Nikon A1R inverted confocal microscope. Migration experiments were interpreted using a modified spot finding algorithm^64^,^13^ in Matlab (Mathworks; Natick, MA). Live-cell 3D migration (Fig. S2) was assessed using data as previously described^13^. Cells were labeled with a cell-tracker dye (CMPTX; Invitrogen), mixed at 5 × 10^5^ cells/mL with DMEM +2.2 mg/mL pH-neutralized collagen-I (BD Biosciences) in glass-bottom multi-well plates (MatTek), polymerized for 30 min at 37 °C, and overlaid complete media overnight. Cells were stimulated 4 h prior to imaging on an environment-controlled Nikon TE2000 confocal microscope for 16 h. Bitplane Imaris software was used to track cells, and MATLAB (Mathworks) was used to calculate the random motility coefficient.

### Computational model of autocrine signaling

We used a multi-compartment ordinary differential equation (ODE) model of autocrine signaling based on previously described implementations^16^,^17^. We built the model to match the geometry of cell-culture experiments in 100 μL wells of 96-well plates, and used two diffusion boundary layers. The first boundary volume (***V***_***S***_) surrounded individual cells with a thickness of 2 μm, the second volume (***V***_***B***_) surrounded the monolayer of cells with a 50 μm thickness, and the third volume (***V***_***BB***_) comprised the remaining bulk supernatant. In the following equations, **Δ** describes diffusion-limited transport through the boundary layers as previously described^16^,^17^. Model terms besides receptor, protease, and bulk antibody or inhibitor concentrations were all initialized at 0. All modeling results were determined from 24 h simulations. Cellular shedding (Fig. 1) was modeled based on the observed HB-EGF productions rates in 12Z. Effects of ligand diffusion (Fig. 2b) were also modeled for HB-EGF, while effects of ligand-receptor binding affinity were modeled for AREG (Fig. 2A). Full model rates (Table S1) and terms (Table S2) are given.

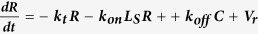
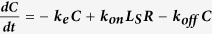
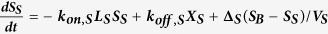
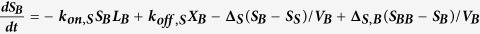
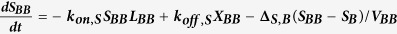
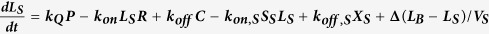
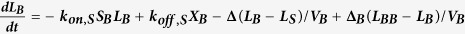
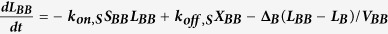
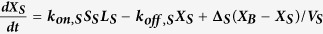
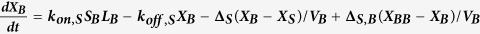
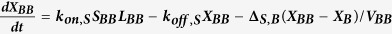
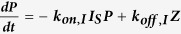
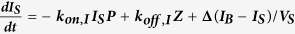
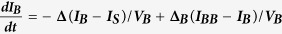
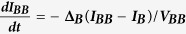

### Correlation analysis of ectodomain shedding and protease activity

Supernatant measurements of endogenous ectodomain shedding and cell surface protease activity were used and expanded upon from previous work, with the addition here of measurements in the presence of mAb225^13^. Recombinant growth factors and cytokines were purchased from Peprotech (Rocky Hill, NJ). EGF was used at a final concentration of 100 ng/mL, NRG1β was used at 80 ng/mL, and all others were used at 50 ng/mL. mAb225 was used at 10 μg/ml, purified from the ATCC hybridoma. For quantification of supernatant substrate accumulation, 12Z were plated on polystyrene plates at 80% confluency, serum-starved overnight, and stimulated the following day with serum-free media supplemented with growth factors after a 30 min. pre-treatment with mAb225. Supernatant was collected 24 h after stimulation, clarified by centrifugation (5 min, 300 × g), and frozen at −20^o^ C for later analysis by traditional ELISA (Duo-set; R&D Systems) or bead-based immunoassay (Widescreen, EMD4BioSciences; Merck KGaA). Cells were immediately trypsinized and analyzed for cell count and viability using ViCell (Beckman Coulter), which was then used for supernatant normalization. Ligand measurements were background-corrected by subtracting the signal obtained from the BB94-treated samples as a negative control. For protease activity measurements, seven soluble, FRET-based synthetic polypeptide protease substrates were added concomitantly with growth factors to serum-starved 12Z cultures, following 30 min mAb225 pre-treatment^13^.

Fluorescence was recorded at five time points, and cleavage kinetics were calculated from the rate of fluorescence increase for each substrate and growth factor condition (n = 4 biological reps.). Protease activity matrix analysis (PrAMA) was used to infer specific ADAM activities from the FRET-substrate cleavage measurements, described previously^26^.

### PA12 expression and purification

Human *ADAM12* was obtained from Origene (pCMV6-XL4/ADAM12). The proADAM12 fragment (R29-K206, 19.9 kDa) was amplified by PCR using the full length ADAM12 gene as template with primers 5′-CACCCGAGGGGTGAGCTTATGGAACCAAG and 5′- GGCTATTTATGCCTTCTTGCCCATGTCTGAG. The PCR product was purified and cloned into pENTR/D-TOPO (Invitrogen). The resulting plasmid, pENTR/proADAM12 (29–206) was used as the basis for expression plasmid construction. The PA12 plasmid was moved into vector pDEST527 (from Dominic Esposito, NCI-Frederick) containing a T7 promoter and an N-terminal 6xHis tag, using LR Clonase II (Invitrogen). Colonies of freshly transformed *E. coli* BL21 DE3 or Rosetta 2 DE3 pLacI, containing plasmid pDEST527/pADAM12 were used in all expression experiments. For expressing Hisx6-proADAM12 as inclusion bodies, 20 mL from an overnight culture grown at 37 °C in LB containing ampicillin was used to inoculate 1 L of LB containing ampicillin. Cultures were incubated at 30 °C with shaking to OD_600_ = 0.6, induced by adding IPTG to 0.2 mM, and grown for an additional 4 h. Cells were harvested by centrifugation for 15 min at 5,500 × g at 4 °C. For inclusion body preparation, 4 g of Rosetta 2 DE3 pLacI bacterial PA12 cell pellets were re-suspended in 25 mL Bug Buster Master Mix (Novagen) containing Complete EDTA-free proteinase inhibitors (Roche Diagnostics) per gram of cell paste. Solutions were rocked at RT for 30 min, centrifuged at 4^o^ C for 10 min at 10,000 × g, and pellets were re-suspended in fresh Bug Master Mix where they were again rocked and centrifuged. From this, purified inclusion bodies were then washed once with 40 mL of 25 mM Tris, pH 8, pelleted, re-suspended in distilled water, and stored at −80^o^ C.

### PA12 purification and refolding

Initial experiments were performed using a 2 mL aliquot of inclusion bodies that was diluted into 30 mL of 6 M urea, 50 mM NaPi, pH 7.6, 5 mM TCEP, and 1 M NaCl (Buffer A) with one tablet of EDTA-free protease inhibitor cocktail (Roche). After solubilization overnight at 4^o^ C, supernatant was loaded onto a 7–10 mL Ni-NTA column equilibrated in Buffer A. The column was then washed with 4–5 column volumes of 15 mM imidazole and eluted with 0.5 M imidazole, both in Buffer A. Protein was concentrated to at least 1–3 mg/mL, and then diluted 1:10 into R.T. refold buffer containing 50 mM CAPS, pH 10, 0.18 M arginine, pH 10, and fresh 5 mM TCEP, pH 9. The solution was rocked at 4^o^ C for 2 days, and then placed in dialysis cassettes (Pierce). After dialysis at 4^o^ C for 24 h in 25 mM CAPS, pH 10, 0.25 M NaCl, and 5 mM TCEP, the material was spun at 4000 × g to remove precipitated protein, dialyzed against 25 mM CHES, pH 9, 0.25 M NaCl, and 5 mM TCEP, re-clarified by centrifugation, and then frozen at −80^o^ C for further use. Preparatory procedures were later improved, yielding improved inhibitor potency (see *SI Methods*).

### Inhibition assays with recombinant enzyme

Recombinant ADAM-10, -12, and -17 were purchased from R&D Systems; ADAM12 was activated by furin as described by the manufacturer. FRET-based synthetic peptide substrates were used to measure recombinant enzyme activity in 96-well plates (Grenier). ADAM12 assays were performed using 15 μM of the FRET-peptide Dabcyl-LAQAhomopheRSK(FAM)-NH2, 25 mM Tris, pH 8, 10^−3^% Brij-35, 10 mM CaCl_2_, and 15 μM furin inhibitor 1 (EMD) at RT. ADAM-10 and -17 were assayed as described previously^65^. IC_50_ values were interpolated from a 6-point dilution curve of PA12, which was incubated with 5 nM recombinant ADAM12, 10 min prior to mixing with the FRET-substrate solution. These IC_50_ values were used to estimate binding off-rates in the computational model.

## Additional Information

**How to cite this article**: Miller, M. A. *et al.* Targeting autocrine HB-EGF signaling with specific ADAM12 inhibition using recombinant ADAM12 prodomain. *Sci. Rep.* **5**, 15150; doi: 10.1038/srep15150 (2015).

## Supplementary Material

Supplementary Information
