# Supplementary material for: Targeting autocrine HB-EGF signaling with specific ADAM12 inhibition using recombinant ADAM12 prodomain
Source: Sci Rep. 2015 Oct 19;5:15150. doi: 10.1038/srep15150 (PMC4609913; doi:10.1038/srep15150)
Supplement: Supplementary Information [file srep15150-s1.pdf]

Supplementary Materials:

Targeting autocrine HB-EGF signaling with specific ADAM12 inhibition using recombinant ADAM12 prodomain

Miles A. Miller[1], Marcia L. Moss[2], Gary Powell[3], Robert Petrovich[3], Lori Edwards[3], Aaron S. Meyer[1], Linda G. Griffith[1], Douglas A. Lauffenburger[1]*

1. Massachusetts Institute of Technology, Department of Biological Engineering, 77 Massachusetts Ave., Cambridge, MA 02139
2. BioZyme, Inc., 1513 Old White Oak Church Road, Apex, NC 27523
3. National Institutes of Environmental Health Services (NIEHS), 111 TW Alexander Dr. RTP, NC 27709

*Corresponding author: lauffen@mit.edu

Keywords: ectodomain shedding; metalloproteinase activity; extracellular signaling; systems biology; ordinary differential equation modeling; reaction/diffusion processes; biologic therapeutic design

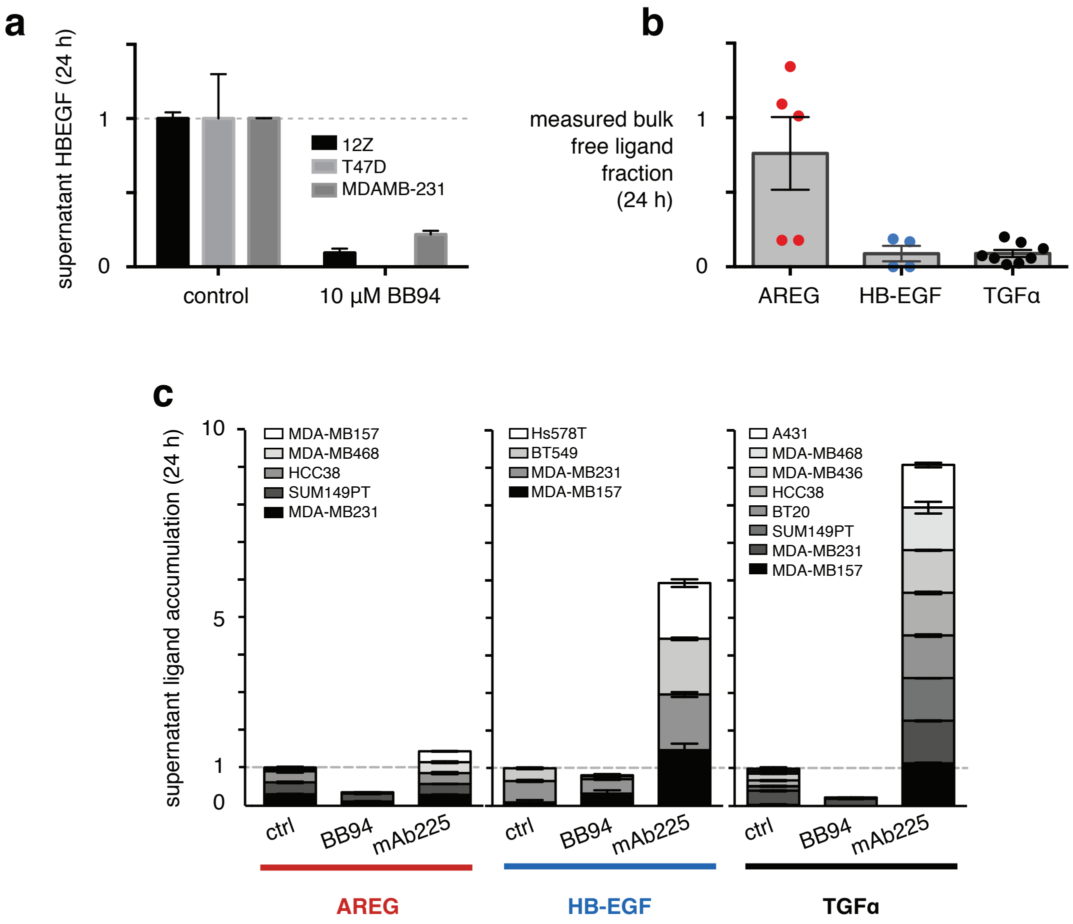

Figure S1. Cancer cell lines exhibit similar ligand capture differences compared to 12Z. a) HB-EGF release is metalloproteinase dependent. Saturating levels of recombinant EGF (100 ng/mL) were used to stimulate accumulation of HB-EGF in three cell lines, which was blocked with addition of 10 µM BB94 5 min prior to stimulation (n=2), plotted as change in concentration from control-treated cultures. b) The fraction of bulk free ligand was experimentally measured by taking the ratio of supernatant ligand concentrations after 24 h with or without the EGFR blocking antibody, mAb225 in a panel of cancer cell lines (each point denotes an individual cancer cell line; n=2 reps). As seen with 12Z (and in agreement with the computational model), the high affinity ligands HB-EGF and TGFα were captured at higher levels compared to the low-affinity ligand AREG. c) Corresponding to data in *b*, supernatant ligands were measured in cancer cell lines in the presence of either BB94 or mAb225. Stacked bars correspond to individual cancer cell lines, presented as a fraction of the summed control values. Results show the mAb225 treatment significantly increases supernatant accumulation of the high-affinity ligands HB-EGF and TGFα, to a greater degree than AREG (n=2 ± SEM).

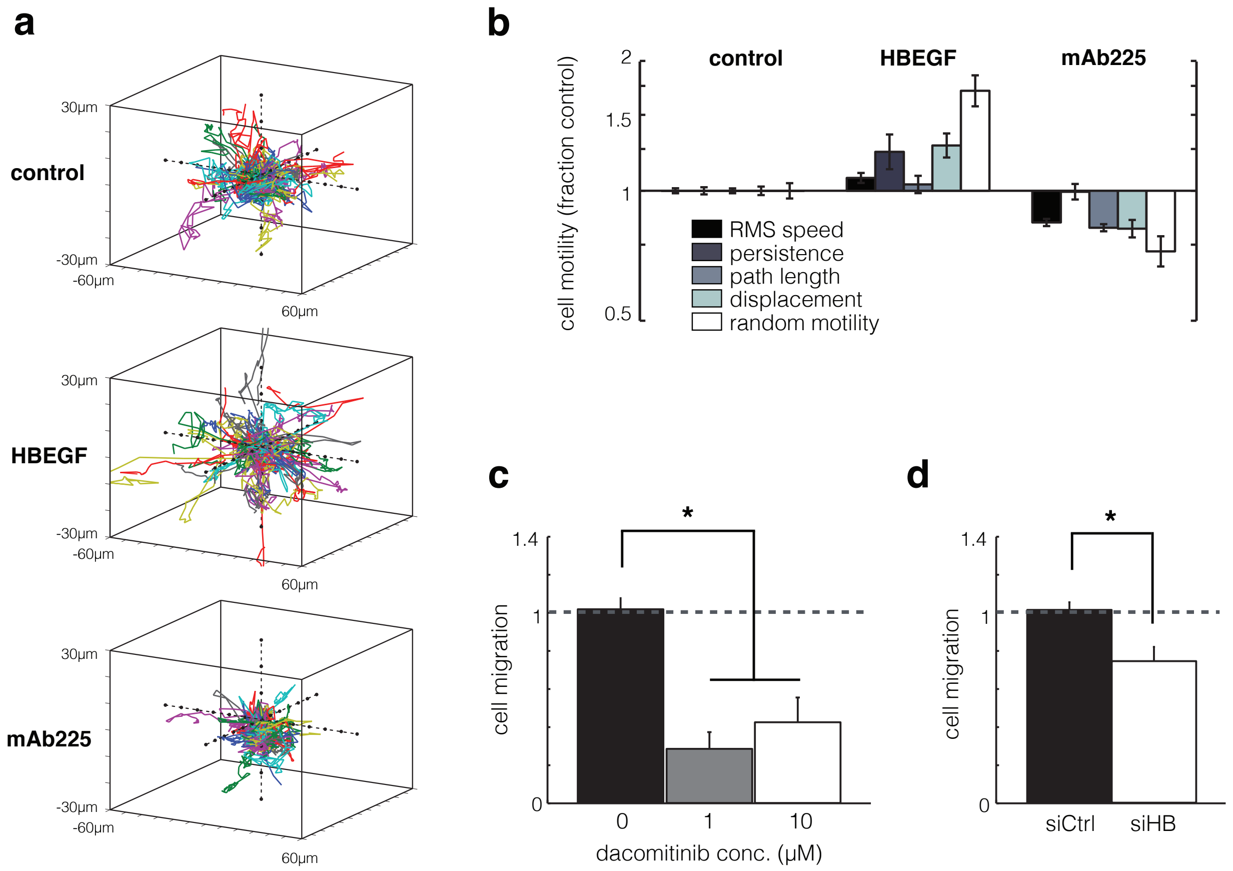

Figure S2. Autocrine ErbB signaling and HB-EGF stimulate cell migration in 12Z. a-b) Exogenous recombinant HB-EGF stimulates enhanced 3D cell migration of 12Z in collagen I gels; treatment with EGFR-blocking mAb225 reduces it (n≥2 experiments; see Miller et al., 2013)[1]. a) Single cells were tracked over the course of 16 h, and their movements are plotted here as individual lines after graphically centering initial positions at the origin. b) Single-cell migration properties corresponding to ***a*** were quantified using a computational model of the persistent random walk. c-d) 12Z cell migration into collagen I gels was quantified after 24 h, using the pan-ErbB inhibitor dacomitinib (c) or siRNA targeting HB-EGF (d) (n≥3 ± SEM; *p<0.05; two-tailed student's t-test).

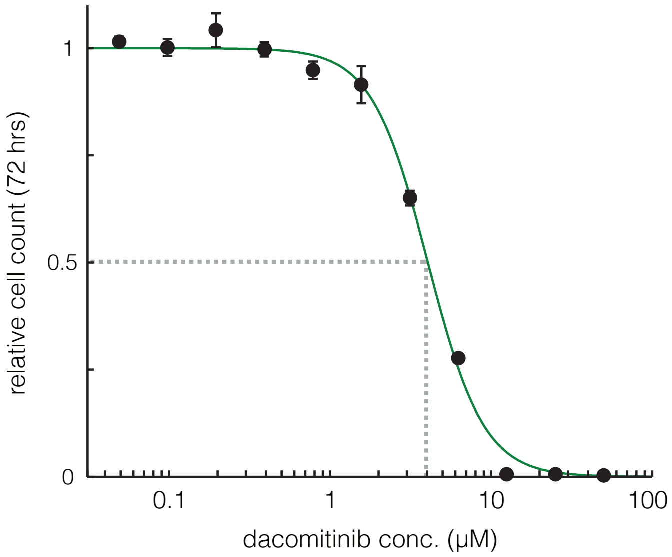

Figure S3. Dacomitinib cytotoxicity in 12Z endometriotic cells. 12Z were treated with dacomitinib for 72 h in a 96-well plate (initial seeding density 5000 cells per well), and cell count was quantified by a resazurin-based assay (PrestoBlue). Dashed line indicates the IC50; n=3 ± SEM. Note the lack of toxicity at 1 µM; this concentration nonetheless impacted cell migration (Fig. S2).

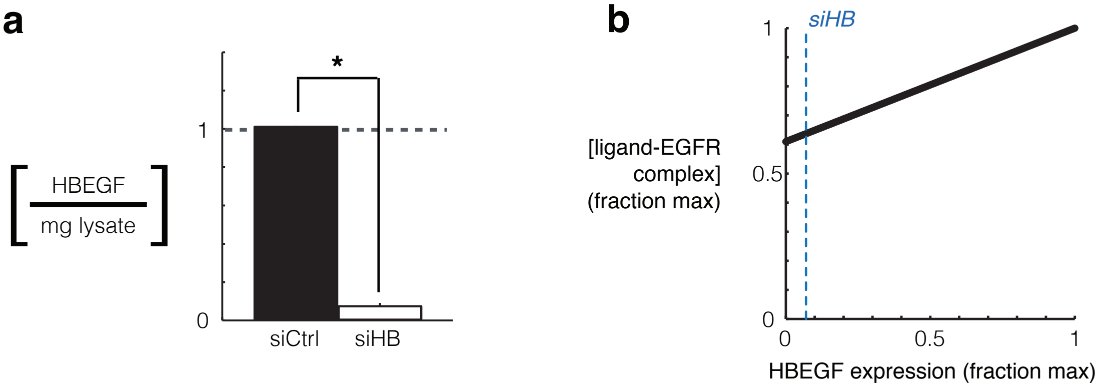

Figure S4. Validation of HB-EGF siRNA knockdown and computational modeling of the resulting signaling effects. a) ELISA measurements of HB-EGF in whole cell lysate show that HB-EGF targeted siRNA significantly reduces protein expression in 12Z (*n≥3 ± SEM; *p<0.05, two-tailed student's t-test). b) Effects of HB-EGF siRNA knockdown were computationally modeled by adjusting the HB-EGF production rate k_Q. The dashed line denotes the fraction of HB-EGF remaining after siRNA treatment (7%), corresponding to *a*. The model predicts HB-EGF siRNA treatment will reduce receptor-ligand occupancy by 36%. EGFR receptor occupancy does not approach zero because the model assumes HB-EGF siRNA has negligible impact on AREG expression and activity.

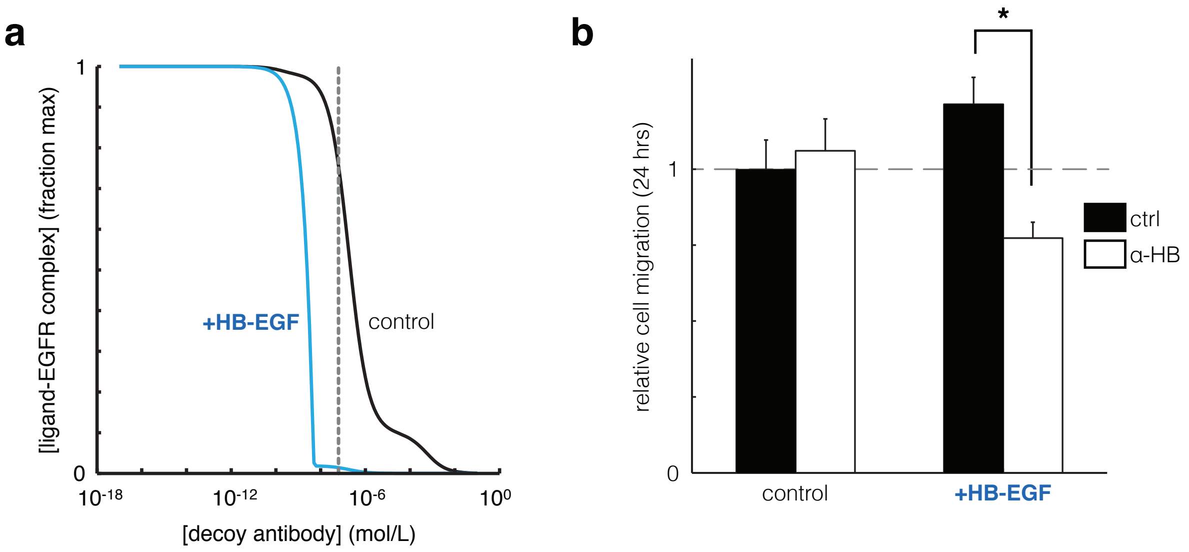

Figure S5. α-HBEGF decoy antibody effectively blocks exogenous HB-EGF signaling but not endogenous autocrine HB-EGF. a) Computationally modeled prediction of decoy antibody efficacy in reducing fractional HB-EGF signaling activity in the presence (blue) or absence (black) of 50 ng/mL recombinant HB-EGF added to the bulk supernatant. Dashed line indicates experimentally tested decoy antibody concentration (10 µg/mL). b) Cell migration experiments confirm modeling predictions that HB-EGF decoy antibody (α-HB) effectively blocks exogenous HB-EGF mediated migratory signaling, but not that of endogenous autocrine HB-EGF (*n≥3 ± SEM; *$p<0.05$; two-tailed t-test).

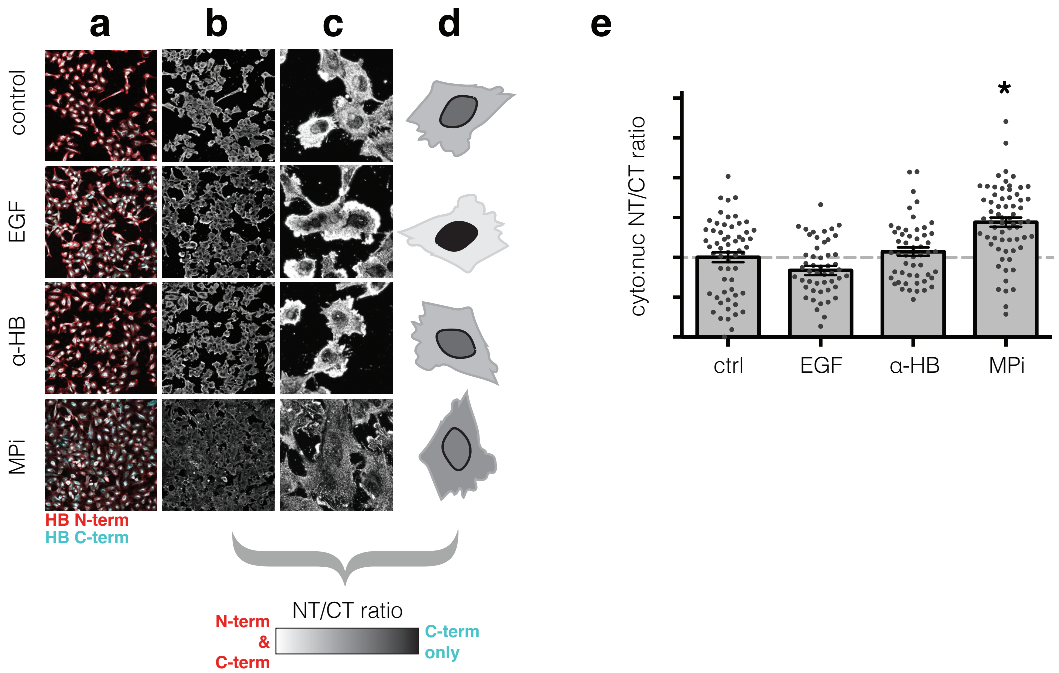

Figure S6. α-HBEGF decoy antibody does not change relative c-terminal HB-EGF nuclear accumulation. a) After 24h treatment with recombinant EGF (100 ng/mL), decoy antibody (10 μg/mL), or the metalloproteinase inhibitor (MPi) BB94 (10 μM), 12Z were fixed, permeabilized, and stained for HB-EGF using N-terminal (NT) and C-terminal (CT) antibodies. b-d) The ratio of NT/CT staining was then calculated, with zoomed-in images highlighted (c) and schematically represented (d). EGF treatment elicited the greatest nuclear accumulation of CT, indicated by dark nuclear regions in the ratiometric images relative to the bright regions elsewhere (c-d). These results suggest nuclear accumulation of c-terminal fragment. In contrast, MPi elicited a significant increase in the nuclear NT/CT ratios, compared to the ratios observed elsewhere in the cell, suggesting lack of c-terminal accumulation selectively in the nucleus. e) Quantification of images from *a-d*, showing decoy antibody treatment does not significantly change nuclear NT/CT ratios compared to the IgG control (n>50 cells / group; n=2 experiments; *$p<0.05$, two-tailed t-test).

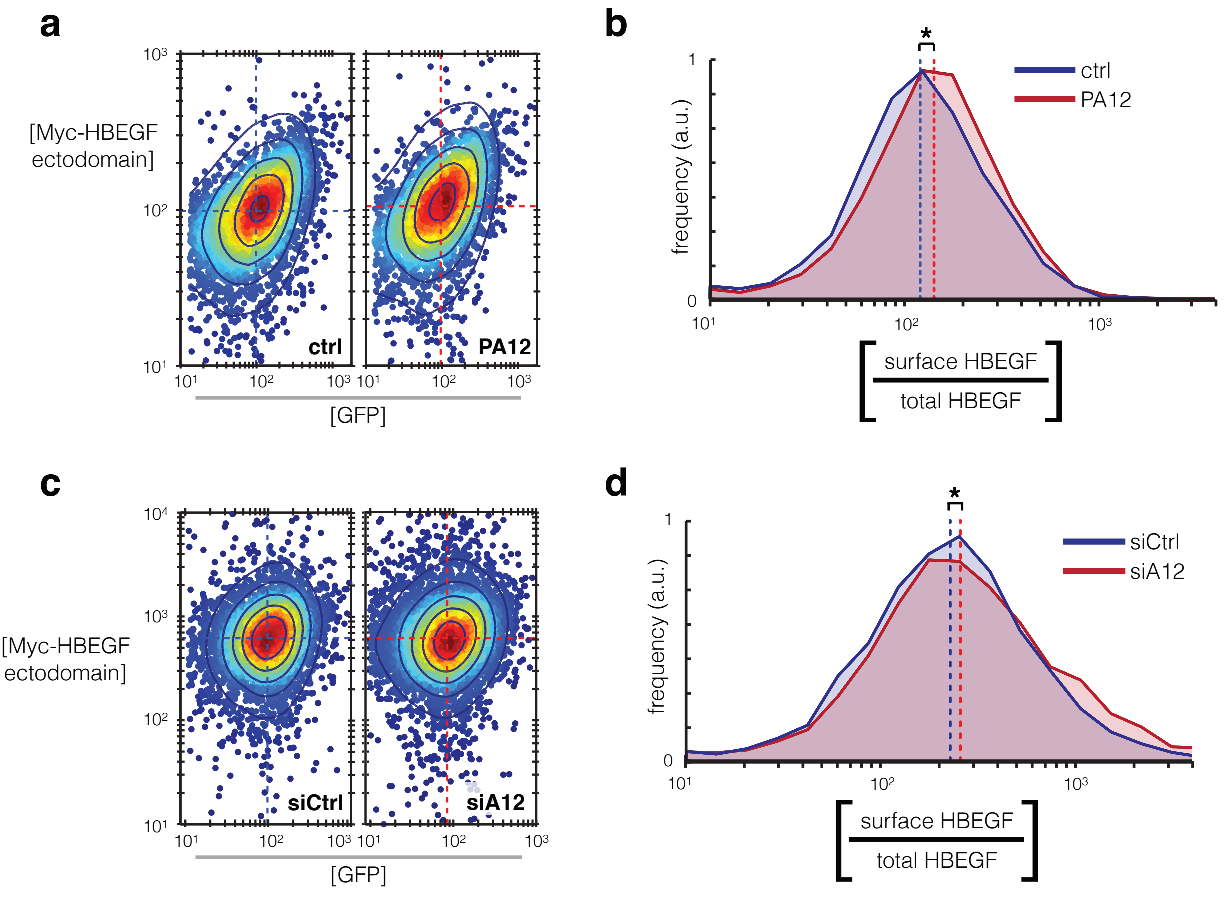

Figure S7. ADAM12 inhibition increases intact pro-HBEGF on the cell surface. 12Z-HE cells stably expressing HB-EGF with Myc-tagged ectodomain and a GFP-tagged cytoplasmic tail were stained, fixed, and analyzed by flow-cytometry. a-b) 2 µM PA12 treatment for 3 h causes an increase in intact transmembrane HB-EGF on the cell surface (corresponding with Fig. 4C). a) Scatter-plot shows the single-cell distribution of Myc staining and GFP fluorescence intensities, with color and contour lines denoting distribution density and dashed lines indicating population medians. b) The ratio of Myc staining to GFP fluorescence intensities was calculated for each cell shown in *a*. c-d) siRNA mediated knockdown of ADAM12 causes an increase in intact transmembrane HB-EGF on the cell surface (corresponding with Fig. 4E). c) Scatter-plot shows the single-cell distribution of Myc staining and GFP fluorescence intensities, with color and contour lines denoting distribution density and dashed lines indicating population medians, after treatment with either a non-targeted control siRNA or an ADAM12-targeted siRNA for 72 h. d) The ratio of Myc staining to GFP fluorescence intensities was calculated for each cell shown in c ($n \geq 3 \pm$ SEM; *$p<0.05$; two-tailed student's t-test).

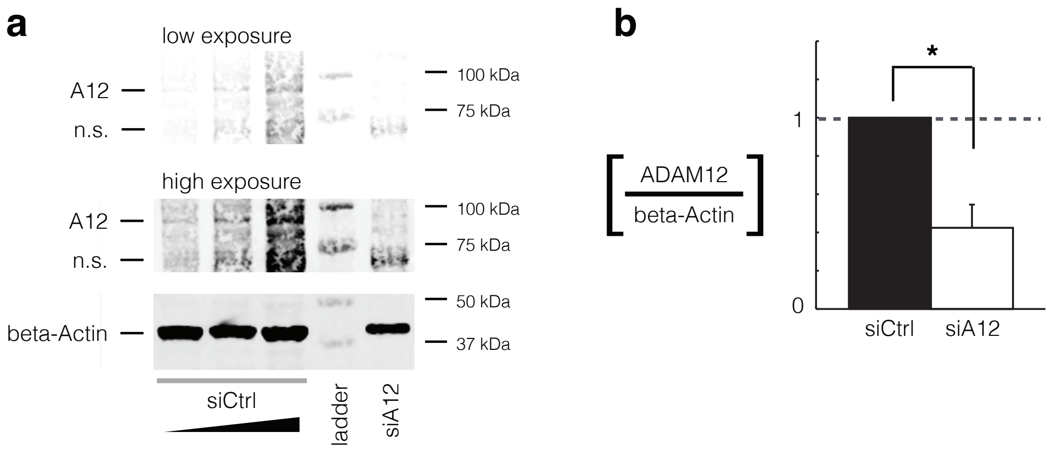

Figure S8. Validation of ADAM12 siRNA-mediated knockdown. a-b) Western blot (a) shows ADAM12-targeted siRNA decreases ADAM12 in whole cell lysate, quantified in ***b***. n.s. denotes non-specific band. *n≥3 ± SEM; *p<0.05, two-tailed student's t-test.

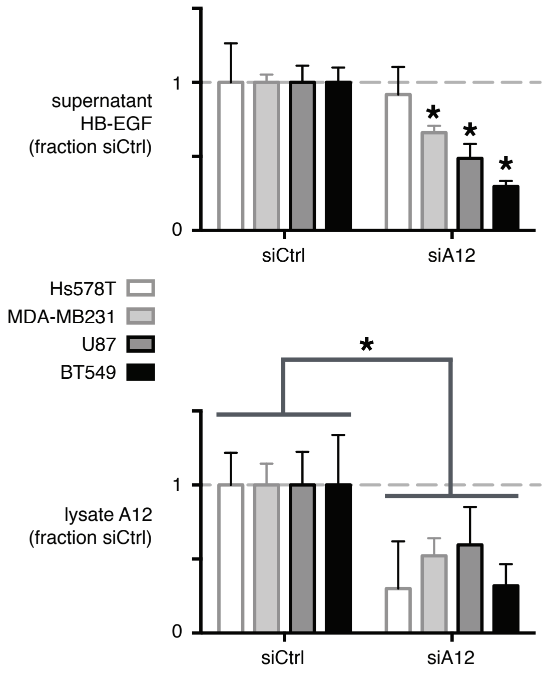

Figure S9. siRNA knockdown of ADAM12 reduces HB-EGF accumulation in cancer cell lines. a) Supernatant accumulation of HB-EGF in the presence of mAb225 was measured following treatment with ADAM12-targeted or scrambled siRNA (24 h post-mAb225; 72 h post-siRNA). Results show ADAM12-targeted siRNA reduces HB-EGF accumulation in 3/4 cancer cell lines tested (*p<0.05; two-tailed t-test; n=3 ± SEM). After removing supernatant for analysis, cells were lysed and analyzed by ELISA to validate ADAM12 knockdown (p=0.0008, pooled t-test, n=4 ± SEM).

	term description	AREG	HB-EGF	Reference
**MW_L (kDa)**	ligand molecular weight	28	22	
**R_0 (receptors • $cell^{-1}$)**	# surface EGF receptors	500,000	500,000	*see methods*
**$k_{Q, basal}$ • P_0 (ligand • min^{-1} $cell^{-1}$)**	soluble ligand production rate	2.3 ± 0.2	0.8 ± 0.1	*see methods*
**$k_{Q, stim}$ • P_0 (ligand • min^{-1} $cell^{-1}$)**	soluble ligand production rate	9.1 ± 0.1	3.1 ± 0.1	*see methods*
**$K_{D, EGFR}$ (nM)**	ligand/EGFR dissociation const.	350	7.1	Sanders et al., 2013
**k_{on} ($M^{-1}s^{-1}$)**	ligand/EGFR binding on-rate	$6.3 \cdot 10^7$	$6.3 \cdot 10^7$	French et al., 1995
**k_{off} (s^{-1})**	ligand/EGFR binding off-rate	0.37	0.0075	Sanders et al., 2013
**D_L (m^2s^{-1})**	ligand diffusion coeff.	$5 \cdot 10^{-11}$	$5 \cdot 10^{-11}$	Rosoff et al., 2005; Thorne et al., 2004
**k_t (s^{-1})**	constitutive receptor internalization	0.0004	0.0004	Wiley et al., 1991; Forsten et al., 1992
**k_e (s^{-1})**	bound receptor internalization	0.004	0.004	Wiley et al., 1991; Forsten et al., 1992
**V_r (M/s)**	receptor synthesis rate	$6.4 \cdot 10^{-9}$	$6.4 \cdot 10^{-9}$	Wiley et al., 1991; Forsten et al., 1992
**$k_{on, S}$ ($M^{-1}s^{-1}$)**	decoy Ab / ligand binding on-rate	10^5	10^5	Thurber et al., 2011
**$k_{off, S}$ (s^{-1})**	decoy Ab / ligand binding off-rate	10^{-5}	10^{-5}	Thurber et al., 2011
**D_S (m^2s^{-1})**	decoy antibody diffusion coeff.	$1 \cdot 10^{-11}$	$1 \cdot 10^{-11}$	Jain et al., 1990; Netti et al., 2000
**P_0 (protease • $cell^{-1}$)**	# initial surface proteases	5000	5000	*see methods*
**$k_{on, I}$ ($M^{-1}s^{-1}$)**	protease / inhibitor binding on-rate	10^5	10^5	Thurber et al., 2011
**$k_{off, I}$ (s^{-1})**	protease / inhibitor binding off-rate	0.043	0.043	*see methods*

Table S1. Reaction parameters for the computational model of autocrine signaling. Parameters references with “*see methods*” were experimentally measured in this work (n≥2 ± SEM), otherwise they were sourced from previously published reports.

term	description
V_S	volume around single cell
V_B	volume around cell monolayer
V_{BB}	bulk supernatant volume
Δ	ligand transport term between V_S and V_B
Δ_B	ligand transport term between V_B and V_{BB}
Δ_S	decoy Ab transport term between V_S and V_B
$\Delta_{S,B}$	decoy Ab transport term between V_B and V_{BB}
R	unbound surface receptor
C	bound surface receptor
S_S	soluble decoy Ab within V_S
S_B	soluble decoy Ab within V_B
S_{BB}	soluble decoy Ab within V_{BB}
L_S	soluble ligand within V_S
L_B	soluble ligand within V_B
L_{BB}	soluble ligand within V_{BB}
X_S	soluble ligand/decoy complex within V_S
X_B	soluble ligand/decoy complex within V_B
X_{BB}	soluble ligand/decoy complex within V_{BB}
P	surface ADAM protease
I_S	protease inhibitor within V_S
I_B	protease inhibitor within V_B
I_{BB}	protease inhibitor within V_{BB}
Z	surface protease / protease inhibitor complex

Table S2. Reaction species for the computational model of autocrine signaling. Transport terms (Δ) describing diffusion-mediated transport through boundary layers is described elsewhere [2,3].

Supplementary Methods: We determined that better yields of purified PA12 could be obtained by solubilization with 100 mL of 6 M urea, 50 mM Tris, pH 8, 200 mM NaCl, 5 mM TCEP (Buffer B). For purification, PA12 was loaded onto a 15 mL Ni-NTA agarose column equilibrated with Buffer B, followed by washing with 15 mM imidazole and eluting with 0.25 M imidazole, all in Buffer B. The 9 mg/mL eluted material was diluted 1:6 in 6 M urea containing 50 mM CAPS, pH 10, 250 mM NaCl, and 5 mM TCEP. Then 90 mL of 50 mM CAPS, pH 10, 0.3M NaCl, 0.18 M arginine, and 5 mM TCEP with 1 EDTA-free proteinase inhibitor (Roche) was prepared at R.T. To this, 3 mL of 6 M urea, 50 mM CAPS, pH 10, and 0.3 M NaCl was added first. Then 8 mL of the 1:6 diluted eluted material was added with stirring. After overnight incubation at 4°C, the solution was spun at 4000 x g to pellet the precipitate. The liquid was removed and dialyzed at 4°C in 2 x 30mL 10 kDa cutoff dialysis bags (Thermo), in 2 L of buffer containing 25 mM CHES, pH 9, 300 mM NaCl, 0.1% BME and 0.06 M urea plus 40 mL of a 4 M urea solution in 50 mM Tris, pH 8, 200 mM NaCl, and 5 mM TCEP. Dialysis was performed for 3 h, the bag removed, and the protein concentrated to about 0.2 mg/ml with centrifugal 10 kDa molecular-weight cutoff filters (Amicon; Millipore). Glycerol was added to 10% and the protein was stored at -80°C. Using this preparation technique, inhibitor potency was assessed as described using recombinant enzymes. IC_{50} were determined to be 68.5 nM +/- 19.7 for ADAM12, and >8 uM for ADAM-8, -10, and -17.

Supplementary References:

1. Miller, M. A. et al. ADAM-10 and -17 regulate endometriotic cell migration via concerted ligand and receptor shedding feedback on kinase signaling. *Proc Natl Acad Sci U S A* **110**, E2074-E2083 (2013).
2. Forsten, K. E. & Lauffenburger, D. A. Autocrine ligand binding to cell receptors. Mathematical analysis of competition by solution “decoys”. *Biophys J* **61**, 518-529 (1992).
3. Forsten, K. E. & Lauffenburger, D. A. Interrupting autocrine ligand-receptor binding: comparison between receptor blockers and ligand decoys. *Biophys J* **63**, 857-861 (1992).
